# Introduction: Re-Configurations: A New Conceptual Framework for Research on the MENA Region

**DOI:** 10.1007/978-3-658-31160-5_1

**Published:** 2020-10-14

**Authors:** Rachid Ouaissa, Friederike Pannewick, Alena Strohmaier

**Affiliations:** 6 Marburg, Hessen Germany; 7 Marburg, Hessen Germany; 8 Marburg, Hessen Germany; 9 Centrum für Nah- und Mittelost Studien, Philipps-Universität Marburg, Marburg, Germany; 10 Institut für Medienwissenschaft/Centrum für Nah- und Mittelost Studien, Philipps-Universität Marburg, Marburg, Germany

## Abstract

This essay collection is the outcome of interdisciplinary research into political, societal, and cultural transformation processes in the Middle East and North Africa (MENA) region at the Philipps-Universität in Marburg, Germany. It builds on many years of collaboration between two research networks at the Center for Near and Middle Eastern Studies: the research network “Re-Configurations: History, Remembrance and Transformation Processes in the Middle East and North Africa” (2013–19), funded by the German Federal Ministry of Education and Research (BMBF), and the Leibniz-Prize research group “Figures of Thought | Turning Points: Cultural Practices and Social Change in the Arab World” (2013–20), funded by the Deutsche Forschungsgemeinschaft (DFG). Both research projects’ central interest lay in the political, social, and cultural transformation that has become especially visible since 2010–11; we conceptualize this transformation here using the term “re-configurations.” At the core of the inquiry are interpretations of visions of past and future, power relations and both political and symbolic representations.

This essay collection is the outcome of interdisciplinary research into political, societal, and cultural transformation processes in the Middle East and North Africa (MENA) region at the Philipps-Universität in Marburg, Germany. It builds on many years of collaboration between two research networks at the Center for Near and Middle Eastern Studies: the research network “Re-Configurations: History, Remembrance and Transformation Processes in the Middle East and North Africa” (2013–19), funded by the German Federal Ministry of Education and Research (BMBF), and the Leibniz-Prize research group “Figures of Thought | Turning Points: Cultural Practices and Social Change in the Arab World” (2013–20), funded by the Deutsche Forschungsgemeinschaft (DFG). Both research projects’ central interest lay in the political, social, and cultural transformation that has become especially visible since 2010–11; we conceptualize this transformation here using the term “re-configurations.” At the core of the inquiry are interpretations of visions of past and future, power relations and both political and symbolic representations.

With the concept of “re-configurations,” building on the work of Norbert Elias, we seek to construct a conceptual framework for notions of figuration processes (Elias 2003) and interdependencies between individual state and non-state actors and groups as well as the resulting restructuring of power relations. The concept describes the inextricable, ever-shifting entanglement between humans and history. We do not take stagnation as a premise (Bar 2009; Rougier 2005). Quite the contrary, the social groups and individuals in the MENA region are situated within continuous dynamics of repositioning. The various interdependencies between social groups and norms, along with the influences of indirect (regional and global) entanglements in regard to economic and social contexts and discourses, lead to a constant re-configuration and reinterpretation of power relations.

The notion of re-configurations expands upon conventional approaches of transformation and transition, which assume that social upheavals are linear and normative, and adapts them to the political, economic, and cultural realities of the MENA region. Michael Mann (1998) has already pointed out that the idea of a “great revolution”—such as the English Revolution, the French Revolution, and the Russian Revolution—, which assumes a clean break between old and new systems, is one of the academic fallacies of historiography. We therefore view the events of 2009–13 in the MENA region, as well as the uprisings in Sudan, Algeria, Lebanon, and Iraq since 2019, as open-ended processes (Bozarslan 2015; Gerges 2014; Dawisha 2013).

The Green Movement of 2009 in Iran and the “Arab Spring” in Tunisia, Morocco, Egypt, Lebanon, Libya, Syria, Bahrain, and Yemen (2011–13) unleashed a deluge of international research into the backgrounds, effects, and meanings of popular protest movements and subsequent developments in the MENA region. In this complex and disparate research discourse, there is a gradually emerging epistemological shift in thinking across disciplines studying the MENA region.

The popular insurgency movements in the MENA region posed a genuine challenge to the social sciences and humanities (Beck 2013). Aside from the discussion as to whether these are upheavals or revolutions (see Bayat 2013; Jünemann and Zorob 2013), these events were embedded in a variety of historical and social trends and contexts. Bozarslan (2015) considers the popular insurgency movements to be the commencement of a new cycle in the history of the MENA region whose outcome remains to be seen. Ajami (2012) calls this the third *Nahḍa*, alluding to the Arab awakening and entry into modernity in the late nineteenth and early twentieth centuries, while Dawisha (2013) calls it the second *Nahḍa*. Unlike Ajami, who counts the 1950s and 1960s—shaped by national leaders such as Gamal Abdel Nasser in Egypt, Habib Bourghiba in Tunisia and the early leaders of the Baath Party in Iraq and Syria—as the second *Nahḍa,* Dawisha only applies the word to the historical renewal movement of the nineteenth century and the upheavals of 2011. Other approaches interpret the events as the end of postcolonialism (see Tripp 2013; Lynch 2013; Khosrokhavar 2012; Dabashi 2012; Perthes 2015).

Likewise, the reasons for the emergence of the popular insurgency movements are interpreted variously. Among the underlying causes researchers point to are the economic policies of postcolonial elites in the MENA region (see Heydarian 2014); the structural weakness of the highly pension-dependent and scarcely diversified regional economies and the associated difficulties with transitioning to capitalism (see Achcar 2013; Hanieh 2011); but also the pressure to conform triggered by neoliberal globalization and its attendant crises, which have also led to popular insurgency movements in other regions of the world (such as the ¡Democracia Real YA! movements in Latin America, the Occupy movement in the US and Europe, and Movimiento 15-M in Spain; see Heydarian 2014; Guazzone and Pioppi 2012). According to other approaches still, these movements resulted from a fragile alliance between a culturally, ideologically, and economically heterogeneous middle class facing decline and a broad, marginalized subaltern stratum (cf. Kurlantzick 2013; Kandil 2012) on the other. Factors such as demographic change (see Courbage and Todd 2007; Ferguson 2011), generational conflicts (see Dhillon and Yousef 2009), and changing gender relations (see Moghadam 2003) also play a role, as shown by the focus on youth cultures in the media and academia (see Honwana 2013; Jung et al. 2013; Wessel 2013; Khalaf and Khalaf 2011). Embedded in the global framework of a transnational, anti-system protest movement and as a result of the structural crisis of global capitalism (cf. Lawson 2012; Wallerstein 2011), the popular insurgency movements in the MENA region (see Beinin and Vairel 2011; Hinnebusch 2014) were ascribed a new collective self-confidence through the intensive use of novel social media platforms (see Jenkins et al. 2013; Strohmaier and Krewani 2021). Meanwhile, other approaches refer to the “Arab winter” (see Abdul-Hussain et al. 2012) in light of wars and the progressive erosion of a number of states in the MENA region due to the rise of political Islam (see Bokhari and Senzai 2013; Cesari 2014; Hamid 2014).

Developments since 2010–11 have contributed to a growth in the reception of a number of research areas within MENA regional studies that had previously been given fairly scant attention. The intervening years have also brought changes in analytical perspectives. For example, political scientists and historians of the MENA region are now devoting greater focus to civil society actors and subaltern groups (Al-Zubaidi et al. 2013; Harders 2015; Cronin 2012). There is also a growing emphasis on the agency of marginalized groups such as women (see Langohr 2015; Khalid 2015), young people (see Abdalla 2016; Ouaissa and Gertel 2014) and ethnic-religious minorities or middle classes blocked from advancement (Ouaissa 2013). In this context, the humanities, social sciences, and cultural studies are more closely considering day-to-day practices, subversive forms of resistance, mobilization techniques, and forms of representation (see Carapico 2014). The uprisings were also interpreted as a rewriting or renegotiation of the social contracts between the states and societies of the region (see Bouziane et al. 2013).

This development is accompanied by the growing influence in research of an expanded notion of politics that treats languages, art, literature, performance, graffiti, music, and film as analytical categories and even as reflections and seismographs of social and political change (Salime 2015; Tripp 2012; Mehrez 2012; Dabashi 2012, p. 225 f.). Here, for example, the focus is placed on linguistic and aesthetic self-expression and the history of its interpretation (see Khalil 2011; Harb et al. 2013; Baker 2015). Some approaches look for portents of popular uprisings in literature (Sakr 2013; Mvogo 2012; El Maarouf 2014), while others examine the role of artistic forms of expression in transformation processes (see Amine and Roberson 2013; O'Rawe and Phelan 2016; Strohmaier 2019; Pannewick 2020).

These popular uprisings have made an important contribution to a new flourishing of efforts to redesign and reclaim public space (see Schielke and Winegar 2012; Kraidy 2016). Visual, auditory, and pictorial cultural practices are regarded as new forms of resistance; in particular, graffiti as a resistance practice and an aesthetic form of expression in public space has received special scrutiny in many publications (see Zoghbi and Don Stone 2011; Hamdy et al. 2014; Gröndahl 2013). Moreover, the popular insurgency movements have raised the question of how to deal with past injustices. Transitional justice processes (see Fisher and Stewart 2014) and memory cultures can be identified in this context as a discursive field in which power struggles over the interpretation of history play out and new, at times self-directed, narratives are shaped.

## Re-Configurations as Dynamic Processes of Change

The concept of re-configurations builds on Norbert Elias’s notion of figuration. Elias uses figuration to describe the foundations of social life in human interdependencies, i.e. a social community in the sense of a social network of mutually dependent individuals (e.g. family, household, club). He argues”that human beings should be seen primarily as interdependent, forming figurations or networks with each other which connect the psychological with the social, or habitus with social relations” (Van Krieken 2017). The term re-configurations extends this concept, breaking away from transition studies. It refers to dynamic change processes within structural, spatially situated, discursive, and symbolic power networks. By taking into account the interaction between action and structure, it makes visible the specific “order of change” (Elias 1978, p. 149).

The concept of re-configurations rests on a conceptual architecture that illuminates the constitution, modification, and transformation of political, economic, social, and cultural relations leading to the formation of new power relations. This paradigm frames patterns of interaction as a mixture of conflict and cooperation, which—going beyond Elias’s understanding of configuration—either usher in new constellations of order or seek to uphold the old ones. If the president of a country is deposed by a coup d’état, as in Egypt, this does not necessarily bring profound systemic change. On the contrary, according to the findings ten years after the popular insurgency movements in the MENA region, a constellation of order can be sustained at its core in spite of superficial corrections.

We therefore understand re-configurations of political, social and cultural systems as a process of both conflictual and cooperative interactions aimed at restoring or even maintaining the old order by building new alliances, whereby the orders, structures and discourses are then modified in order to preclude major changes. Thus, the process-based concept of re-configurations implies negotiations and struggles over societies’ visions of the future, on the basis of historical experience, during the intermediate phase between a period of upheaval and a period of new order. This opens up a perspective for debates concerning participation and political and symbolic representations. The visions of the future are themselves based on historically grounded narratives that shape the renegotiations of political and economic conditions; to paraphrase Koselleck (1988), they are the past of the future.

This essay collection deals with interdependent, overlapping, repetitive, cumulative, and transformative processes (Boudon 1979) whose analysis makes it possible to account for both repetitive structures and dynamics of change (Koselleck 2000, p. 12 f.). By departing formerly dominant narratives of nationalism and Islamism in the MENA region, with their highly ideologized view of history, scholars incorporate a number of heretofore marginalized voices and histories into the broader social discourse. In this way, phenomena that have so far been neglected by research, such as labor disputes, everyday acts of resistance, (semi-)autonomous social spaces and actors, and minority or subaltern groups are brought into the spotlight in order to lend historical depth to research on current developments in the MENA region. For example, Rachid Ouaissa’s chapter analyzes the re-configurations of the Algerian political system. He explains the (re-)establishment of power alliances and traces power shifts through oil price fluctuations on the global market, laying out the concomitant instability of systems of co-option based on the distribution of rent. In times of power crises, the state class is prepared to make concessions, such as economic and political liberalizations. During the 1980s oil crisis, the single-party system was terminated, elections authorized, and the economic system liberalized. Eventually, the civil war (1992–2002) contributed to restoring the system and enabled those with power to co-opt the middle classes anew and secure loyalty to the Bouteflika system through the distribution of rent. Since February 2019, however, an unprecedented mass social mobilization has been underway. The Hirak movement disrupted the order within the state class and forced President Bouteflika to step down, but the regime, under military leadership, still tried to re-configure the political system by eliminating old clans and striking new alliances. This is the story of a political system’s re-configurations that seek to sustain the old order by building new alliances.

The anthology also examines contemporary re-configurations of cultural memory in various countries of the region arising from the weakening of once-hegemonic grant narratives and the corresponding authoritarian systems. Over the past two decades, Germany has seen a significant rise in the study of cultural memory, remembrance cultures, forms of passing down historical knowledge in private and collective contexts, the relationships between public and private memory, the politics of history, etc.

To date these flourishing fields remain underdeveloped in reference to the countries of the MENA region (Silverstein and Makdisi 2006). Yet political upheavals often provoke an increased societal interest in historical subjects and diverse activities for the sake of reconstructing cultural memory. Various actors’ struggles over the power to assert an interpretation and define a society’s cultural memory invite conclusions about the depth of the social and political transformation in the MENA region. In this context, Susanne Buckley-Zistel’s chapter asks what processes of reckoning with the past have been initiated and how these processes relate to the search for justice and the quest for remembrance on a more global scale. In the aftermath of the “Arab Spring,” the affected countries are going through various forms of transitions, significantly re-configuring the MENA region. A number of new civil society actors, political elites and international norm entrepreneurs are engaging with the lengthy histories of repression in the respective countries, as well as with the violence that occurred during the Arab Spring, in order to deal with the legacy of human rights abuses (Sriram 2017). These transitions to justice are not without obstacles and challenges, however. The objective of Buckley-Zistel’s chapter is therefore not to tell a story of various transitional justice and memory projects in post-Arab Spring countries but to situate the practice of doing so in time and space. On a related topic, Perrine Lachenal examines the notion of “martyr” as socially constructed and contested along gendered and political lines in her chapter examining how heroes and martyrs have been produced and deployed in post-revolutionary Tunisia. It begins by revisiting governmental attempts, launched soon after the revolution, to monopolize and institutionally define who could benefit from official recognition as a martyr. Lachenal unpacks the divergent definitions of “martyrdom” according to official institutions and bereaved families respectively, arguing that the boundaries of “martyr” as a moral category are often drawn in terms of differing masculinities. Her chapter goes on to demonstrate how the category of “martyrs of the nation” has progressively overshadowed the category of “martyrs of the revolution” in official memorial practices, as the commemoration of the revolution has progressively focused on its uniformed victims, leaving out the civilian dead.

The judicial dimensions of the political upheavals in the MENA region comprise another key aspect. In the case of Tunisia, the process of transitional justice was carried out after the successful overthrow of the old autocratic order. The Moroccan case shows that a reckoning with the past can also be enacted as a public performance and exploited to justify a new regime. In another sense, contemporary trends in the MENA region offer diverse entry points for transition studies that go beyond judicial reckoning processes. Mariam Salehi’s chapter explains the development of the Tunisian transitional justice process. Drawing on Norbert Elias’s ideas about social processes, she argues that dynamics of transitional justice processes cannot be understood solely via international norms and the “justice industry,” which jointly shape institutionalized transitional justice projects, nor solely by considering the context and political preferences of domestic actors. Rather, she argues, they are shaped by the interplay of planned process with unplanned political and social dynamics; with a political context in flux, power shifts, and sometimes competing planned efforts in other realms. Basing her findings empirically on “process-concurrent” field research in post-Arab Spring Tunisia, she shows that a technocratic/institutionalized transitional justice project can develop dynamics that are somewhat, but not entirely, independent of power shifts. However, the abovementioned interplays may lead to frictional encounters, which trigger feedback loops, new processes, and new structures.

In their chapter, Thorsten Bonacker and Tareq Sydiq explore how, according to scholars of historical sociology, the re-configuration of social and political order in postcolonial society can best be understood as an ongoing (re)interpretation of modernity’s cultural and political program. Thus, diverse actors shape the re-configuration of the social and political order in postcolonial societies and movements that hold competing views on what constitutes a modern society. They argue that one important way of developing these interpretations of modernity is that actors vernacularize ideas and institutional models of modernity from what Bendix has called “reference societies” (1967). A reference society is simultaneously an imagined vision of how a society should look and an existing society to which actors refer when they link their claims for social change to a broader understanding of modernity. The chapter offers, first, a conceptual approach to understand the role of reference societies in the dynamics of postcolonial and post-revolutionary state-building. They argue that especially in situations of transformation and institutional crisis, reference societies function as an important resource for claim-making and identity construction. Second, they illustrate the argument through the case of Iranian society, which has incorporated very different versions of modernity in its postcolonial and post-revolutionary history, making it a particularly interesting example.

Increasingly, however, transformations to states and societies no longer come solely from within. The impact of transregional factors on social processes is growing. Transnational processes are at the foundation of Claudia Derichs’s chapter, which is devoted to a multiregional perspective of a “global 1968.” The year 1968 has a special meaning in some parts of the world, but others do not attach much importance to it. While the European perspective tends to assert a “global 1968,” the timeline may look quite different from another vantage point. The chapter adopts such a vantage point in addressing “1968 and beyond” or the “long 1960s,” as the period is often referred to, as a time of global transformations, but with particular local manifestations in their ideological underpinnings and their justifications for (violent) action. Empirical examples of this include the Middle East and (Southeast) Asia; in particular, the chapter discusses the cases of the June War of 1967 in the Arab world and the massacres in Indonesia in 1965. The defeat of Arab armies against Israel and Indonesia’s tragic events of the 1960s each paved the way for a gradual strengthening of various Islamic missionary and activist movements that spread across both regions and subsequently gained huge mobilizing momentum. This chapter argues that the emergence of strong Islamic movements in this period has had vast repercussions for decades to come, for example by advancing “Islamization” in many countries around the globe. Research on the 1960s should therefore include these movements as substantial change agents in a period of global social and political transformation.

The rise of transregional factors extends beyond the political and artistic sphere; it also includes business. Globally operating entrepreneurs are capable of acting across different economic systems and can either adapt their operations to the local system or contrarily, through lobbying, seek to align the system with their own activities. Steffen Wippel elaborates on northern Morocco and the metropolitan area of Tangier, which has experienced important urban, economic, and infrastructural re-configurations since the late 2000s. This includes the building of a globally significant mega-container port, the reconversion of the old inner-city port area into a glamorous waterfront, the establishment of huge industrial estates and new tourism facilities, and the construction of state-of-the-art land transport routes. Starting from a spatial perspective, his chapter tackles transformative effects on different spatial scales. Notably, it considers large-scale (trans-)regional links emerging from these new megaprojects, which make Tangier an interregional hub and integrate new processes of regionalization on several levels. Furthermore, effects on the city itself concern local processes of territorialization and fragmentation, which also affect people’s daily life in their work as well as leisure activities. In its conceptual considerations, the chapter seeks to grasp the different shifting, intermingling, and interpenetrating scales of effects and interventions with regard to current transformation processes in and around Tangier.

In media and art, we increasingly encounter a transregional audience, in which forms of art and media circulate among regions. This does not only apply to mainstream media and the Internet. Cultural exchange and media fusions also take place around the world at local festivals; the effects of this on a global avant-garde have yet to be seen. The focus here is on how exogenous factors such as international communities and organizations, diaspora groups, and other international actors influence the re-configurations. Alena Strohmaier’s chapter explores the formation of the (self-ascribed) label “Iranian diaspora” and its cinematic representations. The Iranian diaspora and its filmmaking can be used to illustrate the transformation of both diaspora into postdiaspora and diaspora film into postdiaspora film. This re-configuration manifests itself spatially on three levels: the real space of the diaspora, which is subject to socio-political changes; the internal-diegetic spaces in the films themselves, which constantly bring new themes to the fore; and film as its own space-creating entity, which constantly updates its own mediality. In Iranian diaspora film, these different spatial dimensions come together.

Felix Lang’s chapter suggests possible avenues for adapting conceptual frameworks to the study of peripheral cultural production. The events unfolding in Syria since spring 2011 have led to a thorough transformation of the intellectual and artistic space in which Syrian authors, filmmakers, and artists move. Beginning with an overview of the connections of institutions, artists, and works which form this contemporary space of cultural production, his chapter goes on to consider the problems existing theoretical conceptions of such spaces from the sociology of arts (e.g. Bourdieu’s fields or Becker’s art worlds) encounter when faced with the empirical reality of the Syrian case. He shows that the transnational, unstable, and often transient nature of these formations and their links with large-scale socio-political changes, such as wars, are difficult to grasp with conceptual toolkit developed on the model of the exceptionally stable spaces of production of Western Europe and the US. Sihem Hamlaoui discusses the effective implementation of change as a crucial concern for Tunisian educational leaders in the twenty-first century. One of the major challenges to the effective implementation of reforms is the resistance to change among teachers or staff members, as their habits slow the process of implementation of any educational reform. Resistance to technology has been found to be a prominent cause of most system failures, especially when witnessed in the very people who are expected to use the technology the most. This highlights the importance of understanding the causes and possible remedies of technology resistance. Studies explored the black box of resistances and suggested a theoretical explanation. Hamlaoui’s chapter divulges the hidden motives behind Tunisian faculty members; resistance towards digitalization and suggest a theoretical implementation strategy in order to reach the intended goals of reform.

Research repeatedly addressed spatial aspects of transformation processes (for example, spatial inequality as a cause of the 2010–11 protests), but it also raises additional questions, which are discussed in terms of socially constructed space as proposed by cultural studies and the social sciences (e.g. Lefebvre 1974, 2006; Schroer 2006). By now, the struggles over future orders in the MENA region have attained an unforeseen level of drama. (Civil) wars, state disintegration, and quasi-state entities such as the “Islamic State” call into question the postcolonial order in places such as the Kurdistan cantons of Syria and the Kurdistan region of Iraq. Andrea Fischer-Tahir’s chapter turns the focus to the Kurdistan region of Iraq, discussing practices of consumption within the context of the neoliberal transformation. The chapter examines representations of everyday life in light of the consumption of meat, specifically chicken. Across class and spatial lines, today’s Kurdish household tends to consider chicken to be integral part of the daily diet. Industrialized poultry farming started in the 1970s within the context of Baathist modernization, whereas in the 1990s, the “production” of chicken had been extremely limited due to the international embargo. However, the boom of international business since 2003 has improved access to chicken for most parts of the population. Nevertheless, neoliberal restructuration of Kurdistan’s economy brought new disorder as it led to total dependency on food imports, including chicken and eggs, whereas Kurdish farmers are unable to compete with mainly Turkish and Iranian players in commodity chains. The chapter considers ways of making this contradiction visible or invisible, respectively, within Kurdish nationalist narratives that both reflect and structure re-configurations of the social order.

This puts to the test the suitability of “MENA” as a concept and raises the question of how we can conduct research in regional studies “that moves beyond fixed ‘spatial containers’ and other established spatial structures” (Wippel and Fischer-Tahir 2018, p. 13). It is now time to focus on entangled spaces that are trans-nationally and transregionally open-ended—transverse macro-areas, border zones, regional subspaces, and (trans-)local contacts and locations—from a multi-scale perspective. In this context, we understand space as a historically constructed product that also grounded in collectively shared representations (designations, symbols, maps, etc.) that can be handed down or questioned intergenerationally. Ideas of space rooted in different eras form a reservoir of reference points for identity, which can be repeatedly reactivated in new configurations, especially when a hegemonic structure falters (Hirschhausen et al. 2015). Instead of treating the MENA region as a supposedly fixed geographical space, we advocate for framing it more accurately as a highly diverse but densely interwoven ensemble of overlapping “arenas” (Green 2014) consisting of changeable geographies of dense social networks of relationships.

Aside from spaces of conflict, our focus is also on the emergence and movement of spaces of economic exchange, spaces where norms, values, and identities are renegotiated through forms of cooperation. A deterritorialized notion of space also encompasses the post-national “intermediate space,” “transitional space,” or “third space” of cultural production (see Bhabha 1994) as well as spaces of memory and cultural memory. It is also important to clarify how the radically changed spatial and temporal modalities of interaction in the digital age serve to structure social negotiation processes.

Generations constitute another aspect of re-configuration processes. Karl Mannheim (1928) defined a generation as a community whose members develop a similar structure of experience and relevance due to major historical events or sociostructural changes and, depending on the generational unit, act differently as protagonists of social change. Thus, individuals create historical configurations and are themselves formed by them (Pilcher 1994). Some chapters in this volume compare interpersonal generational relations and social generational relations in the different social contexts and theorize them more robustly from a transregional perspective. This concerns the societal structuring of actors’ lifespan (transition to adult status, entanglement with social exclusion; “waithood” Dhillon and Yousef 2009), the renegotiation of the collective past through transgenerational transmission and the “(re)invention” of historical narratives and cultural practices as well as the negotiation of political projects that are represented primarily by the younger generation according to structural logic. Ten years after the Arab Spring, which was widely hailed as the outcome of youthful protests, we must especially identify the biographical patterns that guide processes of engagement and disengagement (cf. Fillieule 2001; Hivert 2013). The phenomenon of “youth” is viewed as a psycho-social space of possibility rather than a life stage with clear quantitative boundaries (King 2002). Although this space is derived from the social and familial march of generations, it can only be qualified in terms of the research subjects’ own patterns of interpretation, their own relevance structure, thus their own representations.

Christoph Schwarz’s chapter asks how transitions to adulthood are institutionalized and brokered. He further asks how class, gender, social exclusion, and precariousness are linked to the structure of these transitions and the hegemonic cultural definitions of “youth” and “adulthood.” Have these regimes shifted since the protest movements of 2011? To explore these questions and reconstruct the respective regimes of youth transitions in Morocco and Spain, his chapter contrasts two social movements: the Moroccan *diplômés chômeurs* (unemployed graduates) and the Spanish PAH (Platform for People Affected by Mortgages) housing movement. Amira Augustin discusses a protest movement that emerged in 2007 in South Yemen called the Southern Movement. At first, it was a loose amalgamation of different groups, most of them former army personnel and state employees of the former People’s Democratic Republic of Yemen (PDRY) who were forced from their jobs after the southern faction lost the war of 1994. Because of state security forces’ brutal treatment of protesters, more and more people joined the demonstrations, and the appeals began to change to concrete political demands, such as national independence of the territory that had once formed the PDRY, which in 1990 unified with the Arab Republic of Yemen to form the Republic of Yemen. To understand how the demands of South Yemeni independence survived more than two decades of repression, Augustin’s chapter investigates the major everyday form of resistance in South Yemen. By appropriating hidden forms of resistance, such as intentional and unintentional intergenerational transmission of a counter-narrative, South Yemenis were able to strengthen the calls for independence in recent years. Using examples from participant observations in South Yemen, she discusses how intergenerational transmission has served as an effective everyday form of resistance in the independence struggle in South Yemen.

Nina Studer’s chapter analyzes and contextualizes French characterizations of drinking habits of the colonized in the Maghreb as inherently childlike. French colonial authors often equated the colonized Muslims in the Maghreb with European children. This infantilization of the colonized can be observed in descriptions of what the colonized habitually consumed, as their drinking preferences and habits allegedly revealed childlike behavior. The texts claimed that the Muslim colonized only enjoyed sugary drinks, such as tea or absinthe; that, “like children,” they only copied French bad habits; and that they lacked the mature characteristics that supposedly regulated the danger of overconsumption among French adults. Elites among the colonized also adopted these discourses and placed them into the wider field of descriptive mechanisms that served to dehumanize and degrade the colonized and, as a direct consequence of this, to justify the continued French colonization of the region.

In both conflictual and cooperative negotiation processes, actors communicate through words, symbols, (moving) pictures, narratives, and performances and create meaning through such representations (Hall 1997). As resources of action and organized forms of knowledge, these representations structure the re-configuration of social interaction systems and evolve in the process (Ankersmit 1997; Baberowski et al. 2008). This anthology therefore also focuses on aesthetic representations of rule, resistance, justice, and martyrdom, which, as central themes of political discourse, simultaneously reflect and structure political re-configurations. After all, assignments and negotiations of meaning in social and political space are closely interrelated with aesthetic representation in works of art.

Re-configurations in politics and society are always reflected, questioned, advanced, or subverted in art. In the context of social transformation processes, new forms of literature and film have drawn a great deal of attention in academia and the media since 2011. Through the prism of popular insurgency movements, some authors view contemporary cultural production in the MENA region as both a harbinger and a catalyst of social change (Sakr 2013). The question of art’s relationship to society and politics has been raised with renewed urgency ever since the earliest days of the upheavals (Hyldig Dal 2013; Kraidy 2016). The currency of this question points to an old hermeneutic problem: how can art and the reality surrounding the artwork be considered in tandem? Do social conditions shape artistic works, or do works of art influence their social context? However you phrase the question, it is rooted in a dichotomous understanding of two disconnected spheres. Now as always, there is a tendency to reduce artmaking to a pure reflection of the social relationships surrounding it or to oversimplify it as the medium of a political message. Or, conversely, with an often euphemistic tone, artmaking is ascribed the power of social transformation without any evidence of such power. Our anthology calls into question this line of argument with its basis in a dichotomy of art versus politics. This entails an increased openness of social science approaches to literary and cinematic subject matter, laying important groundwork for a critical examination of the complex of causes and effects in aesthetic production.

Within this context, Walaa Said’s chapter tackles the theme of violent death and its reflections in dystopian novels, with a close reading of Mohammad Rabie’s *Otared* (2014). Although the rates of violence and death in the Egyptian public places have increased dramatically since January 25, 2011, death and mourning have been dismissed as a focus of Tahrir writing, which is inclined to view that eventful day and its aftermath through a euphoric lens. As a counter-response, the rising wave of dystopian novels has flourished to provide a more confrontational attitude toward death as an inherent component of the revolutionary act. For her part, Charlotte Pardey characterizes the post-revolutionary Tunisian literary scene, addressing the ways in which the Tunisian literary establishment wishes for revolutionary events to be reworked in literature. Novels need time: to be written, read, and ultimately recognized as worthy of honors. Some years after the Tunisian uprising of 2010–11, it is time to explore the literary production in their aftermath. Her chapter focuses on novels written in French and Arabic that have found acclaim in the Tunisian literary scene: they were all recipients of the Tunisian prize for fiction, the Prix Comar d’Or, and all of the chosen works deal in one way or another with the 2010–11 uprising. This permits various insights. First, this chapter compares the novels and explores trends such as references to past revolutions and autobiographic reflections. Second, it asks more structural questions about the context of the novels’ production (authors, publishers) as well as about their honorary reception through literary prizes.

To what extent do literature and discourses of theater negotiate norms, values, and rules and critically comment on social re-configurations? This is the subject of the chapter by Friederike Pannewick. She investigates a crucial turning point in the late 1970s work of Syrian dramatist Saʿdallāh Wannūs (1941–1997). This internationally acclaimed author belonged to a generation of Arab intellectuals and artists whose political and artistic self-conception was strongly molded by the question of Palestine. In the late 1960s and early 1970s, a period marked by significant social and intellectual developments triggered by the country’s defeat by Israel in 1967, Wannūs provocatively formulated the lineaments of a politicizing aesthetic, which was to make Arab theater into a vehicle of hope, instigating political reforms and propelling processes of democratization. In 1977, when President Anwar Sadat of Egypt became the first Arab politician to travel on official business to Israel, where he outlined his plans for peace in a speech to the Israeli parliament, a world came crushing down for Wannūs. He tried to take his own life on the night of this momentous event and did not write plays for more than ten years. This chapter shows how the plays he published after this self-imposed silence turned away from a politicized didactic theatre and towards psychological studies focusing on the individual, as well as minority and gender issues, which might be considered part of what Fadi Bardawil has described as an “inward turn” (2013, p. 1).

As the current world situation shows us, major re-configurations of systems of rule and radical social change can also come about through pandemics, major disease outbreaks, and natural disasters (Harper 2018). In his book *Al-Muqaddima,* Ibn Khaldūn (1332–1406), who developed an early groundbreaking theory on historical change, described the plague that reached North Africa in 1348, claiming his own parents’ lives, as an important factor in the social transformation of his era and more generally in the collapse of an empire. In a matter of months, the current coronavirus crisis has brought an accelerated re-configuration of international, national, and local orders. It has tested not only the resilience of global health systems, but the entire social reality with its at times contradictory discourses and norms. For the MENA region, this global crisis is already highlighting palpable social, economic, and political shifts. The response to the crisis and associated developments mark intensive re-configuration process both on a discursive level (for example discourses of gender and religion) and in regard to power relations as they play out within societies (Alijla 2020) and between civil societies and ruling regimes (Grimm and Fehrenbach 2020).

Thus, the history of re-configurations in the MENA region does not end with the singularity of a popular uprising such as the Green Movement or the Arab Spring. The saga continues with the effects of the Covid-19 crisis. Here, the idea of re-configurations again serves as a conceptual foil, an analytical lens, and an epistemic tool for describing a region as permanently subject to processes and flux.
